# Links between accuracy and effectiveness of laboratory medicine equipment: use of the EUnetHTA core model to compare two analyzers by measuring HbA1c

**DOI:** 10.1017/S0266462324000497

**Published:** 2024-12-03

**Authors:** Chiara Di Resta, Chiara Sacco, Mladen Trbos, Massimo Locatelli, Giuseppe Banfi, Rossella Tomaiuolo

**Affiliations:** 1 Faculty of Medicine, Vita-Salute San Raffaele University, Milan, Italy; 2 Laboratory Medicine Service, IRCCS Ospedale San Raffaele, Milan, Italy; 3 IRCCS Galeazzi-Sant’Ambrogio, Milan, Italy

**Keywords:** Health Technology Assessment, Hb1Ac, hemoglobin variants, innovation in laboratory medicine, laboratory medicine equipment

## Abstract

**Objectives:**

In the field of Laboratory Medicine, the evolution of knowledge and the innovation of technologies are the basis of analytical and diagnostic progress, leading to the development of new solutions based on innovative technologies. However, these advances must be accompanied by evidence of appropriateness, diagnostic effectiveness, and organizational efficiency, considering the test’s first impact on patient outcomes.

**Methods:**

The Health Technology Assessment (HTA) is a valid management tool to support Laboratory Medicine professionals in assessing technologies and which is the most appropriate to adopt. This study is an illustrative case of the application of HTA, exploiting the EUnetHTA Core Model, on two analyzers able to determine the glycated hemoglobin (Hemoglobin A1c, HbA1c), the Capillarys 2 Flex piercing analyzer and the HLC-723G11 analyzer in the Laboratory Medicine Service of the IRCCS San Raffaele Hospital (Milan, IT). The main focus is related to potential differences in methods, organizational aspects, and clinical effectiveness of these approaches for measuring HbA1c.

**Results:**

The EUnetHTA Core Model has proven to be the optimal method for HTA in the field of Laboratory Medicine, as it allows to highlight both the peculiarities of the methods on which the analyzers are based and the clinical efficacy of the laboratory test on specific patient populations, considering individual variations in treatment responses, assessing the potential benefits for individual patients or small groups.

**Conclusions:**

This granular analysis helps provide insights into the effectiveness and value of healthcare interventions at the patient level, contributing to evidence-based decision-making in clinical practice and healthcare policy.

## Introduction

Healthcare technologies are rapidly growing and evolving and are of increasing importance in terms of diagnostic-therapeutic efficacy and efficiency of healthcare outcomes. Therefore, optimizing the technology identification, selection, and adoption process is mandatory.

The European Network for Health Technology Assessment (EUnetHTA) defines Health Technology Assessment (HTA) as a multidisciplinary process that summarizes information about the medical, social, economic, and ethical issues related to the use of health technology in a systematic, transparent, unbiased, and robust manner (1).

However, HTA is increasingly directed towards medical devices (2), especially implantable and expensive devices, and less towards diagnostic procedures, as the World Health Organization (WHO) definition indicates: “Health technologies refers to the application of organized knowledge and skills in the form of devices, medicines, vaccines, procedures and systems developed to solve a health problem and improve quality of lives” (3).

In the field of Laboratory Medicine, the evolution of knowledge and the innovation of technologies are the basis of analytic and diagnostic progress (4): supporting diagnostic progress does not only mean developing solutions based on innovative technologies but these must be accompanied by evidence of appropriateness, diagnostic effectiveness and efficiency of organizational aspects. However, the multiplication of technologies in a short period entails the risk of overlapping between the technology already in use and the innovative one, which, outside the intra- and inter-laboratory validation period, leads to organizational irrationalities and an unjustified increase in expenditure (5). To preserve the value of technological innovation, specialists in Laboratory Medicine must be aware not only of the analytical potential of the technologies but also of the impact of the test on patient outcomes. HTA could support Laboratory Medicine professionals in this regard. However, this tool appears to be little used compared to what is applied to assess other health technologies (6). One reason could be attributed to the fact that laboratory testing rarely improves health outcomes directly, but that usually is part of a complex clinical pathway. However, it should be remembered that laboratory tests guide various medical actions and processes (7) and can potentially improve patient outcomes.

In the context of Laboratory Medicine, the adoption of HTA can be applied in the evaluation of technologies such as analyzers and automation systems, as well as for introducing new biomarkers and diagnostic indications (8).

Among the HTA models, the one that has proven to be most suitable for Laboratory Medicine is the EUnetHTA model, as it focuses on the effectiveness of the test on patient outcomes, focusing both on the intrinsic characteristics of the test (e.g., accuracy) and on the proper diagnostic setting in which the test is used (6). This model has been applied during the COVID-19 pandemic to evaluate the diagnostic accuracy of SARS-CoV-2 serological tests (9) and to develop models for different vaccination strategies (10). This illustrative case shows how test accuracy is insufficient to demonstrate the testing’s clinical effectiveness because the testing results must also indicate a change in patient management, which is shown to change health outcomes.

The concept of effectiveness in Laboratory Medicine is becoming increasingly important, considering the potential of diagnostic technologies in terms of processing volume of biological samples, also due to the automation of laboratory instrumentation, which has made it possible to reduce costs and the time to diagnosis (7). Therefore, assessing test effectiveness requires evidence of the effect of tests on patient outcomes contextualized to the diagnostic setting and the clinical reality of the use of the technology.

This study presents the application of HTA to evaluate the possibility of replacing the glycated hemoglobin (HbA1c) analyzer (the Capillarys 2 Flex piercing analyzer by Sebia, Lisses, France) already in use at the laboratory where the study was conducted with a more recent analyzer (the HLC-723G11 analyzer by Tosoh Corporation, Shunan, Yamaguchi, Japan). For this purpose, according to the EUnetHTA model (11), a methodological framework used within the EUnetHTA, the production of HTA was proposed. The model provides a standardized approach to assessing health technologies, including medical devices, pharmaceuticals, procedures, and other interventions. The purpose of the EUnetHTA Core Model is to facilitate the sharing and comparing information across different countries and to promote consistency and transparency in HTA processes (11). A common structure allows member countries to collaborate effectively and contribute to a central pool of HTA information to aid healthcare policymakers and stakeholders in making informed decisions about adopting new technologies in healthcare systems.

## The case study of analyzers for determining HbA1c and hemoglobin profile

### The clinical-diagnostic context

The determination of HbA1c is required to diagnose diabetes mellitus (DM) and, above all, for the retrospective evaluation of patients’ glycemic control, to be performed every 3–4 months (12).

**Figure 1.**
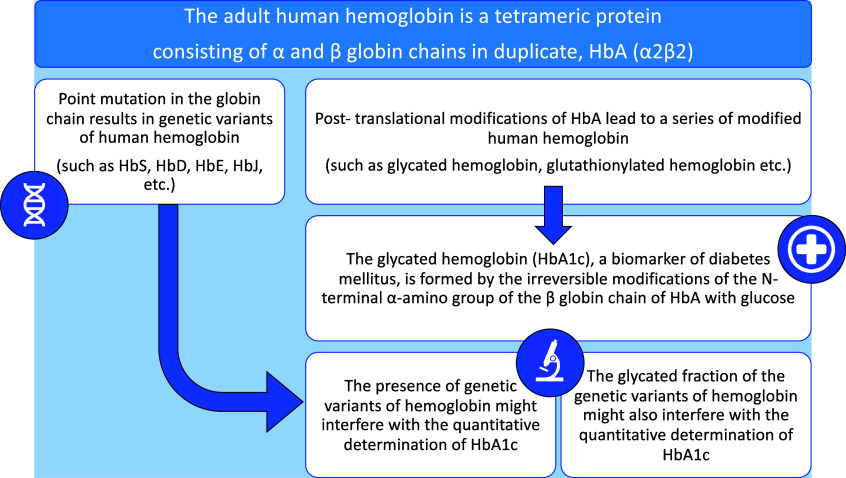
Schematic representation of analytical-diagnostic criticalities in determining glycated hemoglobin (HbA1c). HbA1c is used in the diagnosis and monitoring of diabetes. Still, the presence of hemoglobin variants can interfere with its determination, making the analysis’s results unreliable or difficult to interpret. A hemoglobin variant can cause both positive and negative interference in the measurement of HbA1c. The most frequent cases of interference are due to hemoglobin variants that could not form stable adducts with glucose, could show glycation kinetics different from HbA or could have a reduced erythrocyte average life.

To quantify the HbA1c and to perform a complete hemoglobin profile, it is necessary to separate and quantify the different hemoglobin fractions: both determinations can be obtained using various methods, including ion-exchange high-performance liquid chromatography (HPLC) and capillary electrophoresis (CE) (13). Significantly, hemoglobin variants may interfere with the correct determination of HbA1c (14) (Figure 1). Therefore, in the presence of a hemoglobin variant, the laboratory should verify the effect on the measurement of HbA1c (14).

In the Laboratory Medicine Service of the IRCCS San Raffaele Hospital, around 200 HbA1c analyses are performed every day, six days a week, and approximately fifty hemoglobin profiles are conducted twice a week. The Sebia Capillarys 2 Flex-piercing instrument, based on capillary zone electrophoresis, has been used for years to process a huge volume of routine samples. An opportunity arose to test the Tosoh HLC-723G11 analyzer based on HPLC.

Therefore, the policy question arose of how the Laboratory Medicine Service of the IRCCS San Raffaele Hospital could evaluate the introduction of the Tosoh HLC-723G11 analyzer for the analysis of HbA1c, considering the impact on diagnostic accuracy for DM, efficiency in processing a high volume of routine specimens, and potential implications for patient care. Integrating the new analytical tool into existing laboratory practices while ensuring that diagnostic accuracy, operational efficiency, and overall impact on patient management involve decisions on resource allocation and adjustments to operational protocols within the Laboratory Medicine Service. Therefore, before evaluating the possible replacement of the analyzer in use, an HTA was performed.

Table 1 shows the PICO framework (Population, Intervention, Comparator, Outcome); the related research question is: “Does the Tosoh HLC-723G11 analyzer, based on HPLC, provide a more accurate determination of HbA1c levels and hemoglobin profiles compared to the Sebia Capillarys 2 Flex-piercing instrument in patients with DM, especially in the presence of hemoglobin variants?”

**Table 1.** The key categories of the PICO framework concerning the research question, that is whether the Tosoh HLC-723G11 analyzer was able to provide a more accurate determination of glycated hemoglobin (HbA1c) levels and hemoglobin profiles compared to the Sebia Capillarys 2 Flex piercing instrument in patients with diabetes mellitus (DM), especially in the presence of hemoglobin variants

Population (P)
Patients requiring diagnostic evaluation for DM and monitoring of glycemic control via HbA1c
Intervention (I)
Tosoh HLC–723G11 analyzer based on high-performance liquid chromatography
Comparator (C)
Sebia Capillarys 2 Flex-piercing instrument based on capillary zone electrophoresis
Outcome (O)
Accuracy of HbA1c measurements, considering the presence of hemoglobin variants Operational efficiency, especially with respect to the volume of analyses performed Overall impact on patient care, which could be evaluated through a health technology assessment

## Materials and methods

Among the various HTA models, the authors have chosen the Full HTA, using the EUnetHTA Core Model version 3.0. The structure of the EUnetHTA Core Model version 3.0 includes the following domains: Health problem and current use of the technology, Technical characteristics, Safety, Clinical effectiveness, Organizational aspects, Impact on the patient and society, Ethical analysis, and Legal aspects. The information about the two analyzers was obtained from consulting the manufacturers’ user manuals (15;16), and by searching in the scientific literature (Pubmed from 2013 to 2023; keywords: Capillarys 2 Flex piercing analyzer; HLC-723G11 analyzer), as reported in Prisma Flow Diagram (17) (Supplementary Figure S1). We initially identified seventeen studies, of which all were screened, and eleven were assessed for eligibility and met the inclusion criteria based on relevance, study design, and methodological rigor. To evaluate the quality of the included studies, each study was independently assessed by two reviewers for the quality of execution and clarity of reporting. Discrepancies were resolved through discussion and consensus.

Following the PICO framework, our research question was structured to compare the effectiveness of two HbA1c analyzers in diagnosing and monitoring DM, particularly in the presence of hemoglobin variants. This approach directed our systematic literature review, which adhered to the PRISMA guidelines (17).

Furthermore, the opportunity to have both instruments available and thus be able to carry out use tests at the Laboratory of the IRCCS San Raffaele Hospital has contributed to obtaining helpful information.

## Results

All domains of the EUnetHTA Core Model helped conduct the HTA of the Capillarys 2 Flex-piercing analyzer and HLC-723G11 analyzer.

### The health problem and current use of technology domain

The analysis of HbA1c has a fundamental role in monitoring diabetes and has also been adopted as a diagnostic criterion (HbA1c >48 mmol/mol, HbA1c >6.5 percent) (18). According to the European Society of Cardiology and the European Association for the Study of Diabetes Guidelines, HbA1c is a useful measure of the efficacy of glucose-lowering treatment, is an integrated summary of circadian blood glucose during the preceding 6–8 weeks, equivalent to the lifespan of erythrocytes, should be performed 3–4 times a year (19).

However, the presence of an Hb variant may cause interference in the measurement of HbA1c (14). More than 1400 variants of adult hemoglobin have been described, many of which are clinically silent (20). Around 7 percent of the world’s population is affected by hemoglobinopathies (21). In 2019, there were 6337 cases of thalassemia in Italy and 2023 cases of other hemoglobinopathies (22).

**Figure 2.**
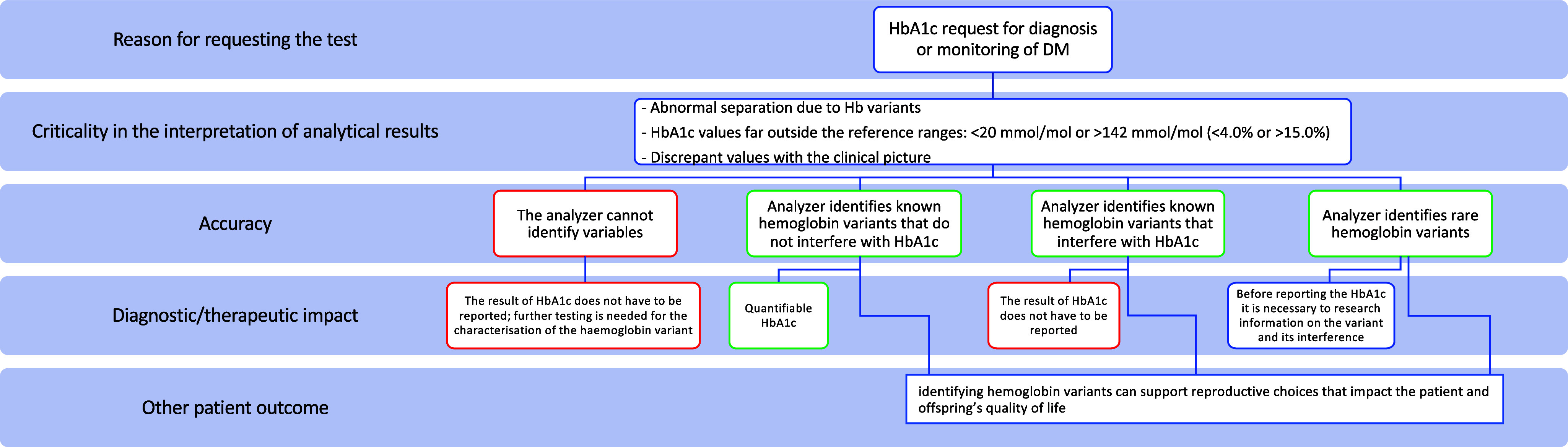
Link between accuracy and efficacy in the determination of glycated hemoglobin (HbA1c) in the diagnostic setting of diabetes mellitus. The use of an analyzer able to identify the different forms of hemoglobin in the presence of hemoglobin variants may have an analytical and clinical relevance for the detection of HbA1c, reducing the risk deriving from incorrect information.

In this context, an analyzer capable of performing in-depth identification of the different forms of hemoglobin in the case of HbA1c results that are suspicious due to the presence of hemoglobin variants (Figure 2) has analytical and clinical relevance, reducing the risk deriving from incorrect information.

### The technical characteristics domain

The analysis of the user manuals and the scientific literature consulted (Supplementary Figure S1) establishes that the analytical method is the main difference between the two analyzers.

The Sebia Capillarys 2 Flex-piercing instrument is an automated analyzer that performs a complete hemoglobin profile for the quantitative analysis of the hemoglobin fractions A, A2, and F and the detection of major hemoglobin variants S, C, E, and D; it is based on the principle of CE in free solution. This technique separates charged molecules by electrophoretic mobility in an alkaline buffer according to the electrolyte pH and electroosmotic flow (23). The hemoglobins, separated in eight parallel silica capillaries, are directly and specifically detected at an absorbance wavelength of 415 nm which is specific to hemoglobins. The resulting electropherograms provide a relative quantification of individual hemoglobin fraction (such as A2 hemoglobin for ß thalassemia diagnostic) and allow the identification of hemoglobin variants (23). Capillary electrophoresis separates the hemoglobin variants based on their surface charge under high voltage. However, depending on the change in surface charge, the glycated fraction of HbA might co-elute with the native fraction of variant hemoglobin, and the glycated fraction of variant hemoglobin might co-elute with the native fraction of HbA, causing an inappropriate estimation of HbA1c (14;23). The Phoresis software allows HbA1c calculation according to the IFCC recommended formula: HbA1c = HbA1c/(HbA1c + HbA0). The results are expressed in both units (IFCC and NGSP).

The Tosoh HLC-723G11 is an HPLC with ion exchange and has as a detection method the absorbance at two wavelengths (415 nm/500 nm). Hemoglobin separation is performed by utilizing differences in ionic interactions between the cation exchange group on the column resin surface and the hemoglobin components in a step gradient elution containing three citric acid buffers with different pH and salt concentrations. The method exploited the surface charges of hemoglobin and, through different retention times, allowed the identification of the Hb variants (14). The results are shown on high-resolution chromatograms. The analyzer works in the standard mode or the variant mode. In the standard mode, it allows the separation of HbA1a, HbA1b, HbF, Labile HbA1c, Stable HbA1c, and HbA0; whereas, in the variant mode, it enables the separation of HbA1a, HbA1b, HbF, Labile HbA1c, Stable HbA1c, HbA0, corrected HbA1c result, in the presence of some Hb variants. HbA1c has a reduced positive surface charge if compared to HbA; therefore, HbA1c is eluted ahead of HbA, resulting in either an overestimation or underestimation of HbA1c values.

### The safety domain

The risks for patients related to the analysis for HbA1c are those of an incorrect diagnosis and an inaccurate quantification with a negative impact on therapeutic decisions (18). Regarding the hemoglobin qualitative abnormalities, there is the possibility of unexpectedly recounting a hemoglobin variant and obtaining a false negative for evaluating hemoglobin structure (24). This can determine a lack of follow-up, the risk of developing a hemolytic crisis and procreative risk.

Both instruments exhibit precision, linearity, and reliability parameters for HbA1c measurement and control of diabetes compliance with various clinical interference conditions routinely encountered in clinical laboratories; in fact, both analyzers have been certified by the NGSP (National Glycohemoglobin Standardization Program) (13), a program aiming to standardize the methods used to measure HbA1c.

Moreover, the analysis of the user manuals shows that no differences were found between the two technologies concerning the safety of healthcare workers linked to the risk of contact with biological material and the hazardousness of the reagents.

### The clinical effectiveness domain

The Laboratory has a fundamental role in recognizing hemoglobin variants. The interpretation of the possible interference on the quantification of HbA1c as accurate laboratory results enables healthcare professionals to make knowledgeable choices in managing DM (25;26). Also, the periodic evaluation of HbA1c allows patients to adapt their lifestyle, contributing to glycemic control and preventing complications. With a positive impact on mortality, morbidity, and quality of life (27).

The accuracy of the two analyzers examined was evaluated thanks to the presence of certifications, the study of the existing literature (Supplementary Figure S1), and the data obtained from evidence of use conducted at the IRCCS San Raffaele Hospital Laboratory Medicine Service. As mentioned, both analyzers have been certified by the NGSP (National Glycohemoglobin Standardization Program) (13).

The direct determination of stable HbA1c was coefficient of variation (CV) <2 percent (28;29) for Tosoh HLC-723G11 and <2.5 percent for Sebia Capillarys 2 Flex-piercing (30;31). A low CV is the result of lower variability among measurements. Small variability of HbA1c values can impact a patient’s journey diagnosis, therapeutic options assessment, disease management, treatment monitoring, and disease progression. Both analyzers show good linearity in HbA1c measurement with a 95 percent CI for the intercept and slope (23;28).

For the Sebia Capillarys 2 Flex-piercing instrument, the evaluation of precision parameters (total error, within-day, between run, and between days) estimation revealed that the precision was within the accepted targets for imprecision (2 percent) based on values expressed by the NGSP system (23;31;32). For the Tosoh HLC-723G11, the repeatability and within-laboratory precision analyzer were within 1 percent (28;29).

From the tests of use carried out at the Laboratory Medicine Service of the IRCCS San Raffaele Hospital, there appears to be a good correlation between the two analyzed (*R* ^2^ = .972) for the HbA1c; instead, around the diagnostic threshold, *R* ^2^ drops to .7032. The determinations of HbF and HbS have a reasonable correlation (respectively *R* ^2^ = .9274 and *R* ^2^ = .9667); whereas, HbA2 shows lower correlation results (*R* ^2^ = .658). These data confirm those in the literature, stating that HPLC and CE are complementary methods and can be used in tandem for accurate and precise hemoglobin variant quantification (28;33;34).

Identifying and characterizing hemoglobin variants during the HbA1c test impacts the reporting and, therefore, diagnostic-therapeutic decisions (test effectiveness), as if a hemoglobin variant is found, it is necessary to check whether this variant interferes with the measurement of HbA1c or not (Figure 2). The most common hemoglobin variants (HbS, HbC, HbD, and HbE) can cause marked hemolytic anemia, and therefore, because the concentration of HbA1c depends on erythrocyte life, it is advisable not to use this biomarker for glycaemic control in these patients. In addition, in some subjects, HbA may be totally missing, with the consequent absence of the glycated form and the possible presence of the glycated component of the variant. Finally, most Hb variants that interfere with HbA1c measurement are rare or clinically asymptomatic (35), their clinical risk should not be underestimated if they coexist with other variants or thalassaemic traits.

### The organizational aspects domain

Both analyzers perform whole or diluted blood (EDTA) analysis, record analytical sessions, can be connected to the laboratory information system, and must be used by Laboratory Medicine personnel trained in the context of a biomedical laboratory.

As HbA1c test results have become more critical and the demand for the test has increased, fast, accurate, and standardized reporting of HbA1c test results has become one of the main challenges. Turnaround time (the testing time from mounting the samples to reporting the results) differed for the HLC-723G11 variant mode from the standard mode (28). In particular, for the HLC-723G11 variant mode, the time required was 3 minutes for one sample, 12 minutes for ten samples, and 31 minutes for thirty samples; instead, for HLC-723G11 standard mode, the time required was 1 minute for one sample, 7 minutes for ten samples, and 18 minutes for 30 samples (28). Instead, Sebia Capillarys 2 Flex-piercing can provide 38 hourly results (36).

A key difference between the two analyzers is that the Tosoh HLC-723G11 is a newly released instrument, whereas the Sebia Capillarys 2 Flex-Piercing analyzer currently in use faces the risk of obsolescence, potentially leading to challenges in maintenance and consumable availability. In the specific context, these last considerations critically impact the cost domain (with a difference of about 70 percent between the quotation of the two instruments).

The organizational impact of adopting new HbA1c analyzers extends beyond mere technical specifications to encompass how well these technologies fit within existing laboratory workflows and meet clinical needs. By systematically evaluating the effectiveness of the HLC-723G11 analyzer compared to the Sebia Capillarys 2 Flex-piercing instrument, we identified not only the superior diagnostic capabilities of the HLC-723G11 but also its operational advantages. These include quicker turnaround times and improved adaptability to high-throughput settings – critical factors in high-volume laboratories where efficiency and accuracy directly impact patient care outcomes. Our findings suggest that integrating the HLC-723G11 could significantly enhance operational efficiency without compromising diagnostic accuracy, especially in detecting and managing diabetes in patients with hemoglobin variants. This evidence-based approach aligns with our organizational goals to optimize resource allocation, reduce redundant processes, and maintain high standards of patient care.

### The impact on the patient and society domain

As with many other laboratory tests, the patient does not perceive technology directly. However, the test improves overall patient outcomes by providing clinicians with a complete picture of a patient’s Hb profile. Indeed, many studies demonstrated that lowering the HbA1c level reduces the complications of diabetes (37). Therefore, systematic HbA1c monitoring is critical in public health management, considering that the prevalence of DM in Italy is estimated at 5.9 percent, with an increasing trend (38). On the other hand, to control the spread of hemoglobinopathies, the identification and characterization of genetic variants of hemoglobin are critical in geographical areas at high risk for hemoglobinopathies and with high migratory flows (31;39).

### The ethical domain

No differences emerged between the two instruments in the ethical. The careful evaluation of an Hb profile can prove extremely useful in increasing the finding, even occasionally, of new hemoglobin variants that, even if not clinically relevant for the patient, can give rise to critical hematological phenotypes when combined with other defects of the beta and alpha chains (39).

### The legal domains

Laboratory tests for DM and hemoglobinopathies are included in the essential levels of assistance, which are the benefits and services that the Italian National Health Service is required to provide to all citizens, free of charge or upon payment of a participation fee (ticket), with public resources collected through general taxation (DPCM January 12, 2017) (40).

A summary of the comparison between the two technologies for each domain is presented in Supplementary Table S1.

## Discussion

From the information presented, it can be concluded that both analyzers have distinctive characteristics and capabilities that must be carefully evaluated based on specific diagnostic needs, especially concerning the presence of hemoglobin variants. The main question concerns the accuracy of determining HbA1c and hemoglobin profiles in patients with DM, particularly in the presence of hemoglobin variants.

As the precision and accuracy of HbA1c measurements are crucial for an accurate diagnosis and diabetes management, a detailed assessment of the technical characteristics, precision, and correlation with hemoglobin variants is essential.

Based on the information provided, both analyzers have passed the National Glycohemoglobin Standardization Program (NGSP) certifications and show good precision in HbA1c measurements. However, it is necessary to carefully consider the strengths and limitations of each instrument, especially concerning the effective management of hemoglobin variants and the impact on operational efficiency in the specific context in which they will be utilized.

Additionally, factors such as turnaround time, laboratory personnel training, instrument maintenance, and the costs associated with using and maintaining each analyzer should also be considered.

Therefore, considering all these factors, a detailed comparative evaluation of the two analyzers will be crucial in determining which instrument might offer a more accurate evaluation of HbA1c and hemoglobin profiles, especially in the presence of hemoglobin variants, in patients with DM.

The EUnetHTA Core Model has proven to be a flexible and adaptable method to evaluate the different aspects of two health technologies at the micro level, i.e., considering the point of view of Laboratory Medicine. In this field, the model developed by EUnetHTA is particularly suitable as it allows the investigation of how the consequences of the tests affect the patient’s outcome. The domain of clinical effectiveness is based on the concept that differences in test accuracy impact clinical decision-making (6). In the analyzed case, the methods play a crucial role. Hemoglobin electrophoresis, the method on which Capillarys 2 Flex-piercing analyzer is based, is a well-established technique routinely used in clinical laboratories for screening samples for hemoglobin profile; however, given the number of hemoglobin variants (20), and despite being mostly uncommon and clinically insignificant, they can interfere with HbA1c measurement. HPLC technology, the method on which the HLC-723G11 analyzer is based, separates the different hemoglobins fractions, so the laboratories using HPLC-based tests provide valuable additional information to clinicians and improve patient care.

An additional advantage of the EUnetHTA Core Model in the HTA of Laboratory Medicine is that it emphasizes the role of the laboratory test in distinguishing between clinical effectiveness evidence and safety (6). Possible interferences related to hemoglobin variants must be carefully considered by the clinician requesting the examination and the laboratory specialist, who must provide a reliable result and an unambiguous interpretation. Thus, it is clear that, in the case of DM and hemoglobin abnormalities, an instrument able to perform a more accurate separative method allows for reducing the risk derived from incorrect clinical information addressed to the patient. Therefore, it would be preferable to adopt methods capable of reporting the presence of abnormal hemoglobin fractions so that the laboratory professional is alerted in evaluating the hemoglobin profile (Figure 2).

EUnetHTA Core Model emphasizes the importance of evaluating safety as a type of outcome in its own right: in this case study, the direct harms of the test are related to imprecise quantification with a negative impact on therapeutic decisions, whereas the “increased safety” as a result of better accuracy, concerns the possibility of unexpectedly recounting a hemoglobin variant.

Finally, the EUnetHTA Core Model focuses on the organizational aspects (6). The prevalence of DM, especially type 2, is continuously increasing: 61 million adults are reported affected in 2021 in Europe, and it is expected that in 2030, this number will grow to 67 million and reach 69 million in 2045 (41). Therefore, increased requests for HbA1c are easily conceivable, becoming a critical marker in Public Health Management. Consequently, it will be essential to have analyzers available that are safe and accurate from a clinical point of view but also efficient from an organizational and economic point of view.

HPLC-mass spectrometry (MS) and CE (6) are reference methods for HbA1c, according to the International Federation of Clinical Chemistry and Laboratory Medicine (IFCC). Still, these assays are limited because of cost-effectiveness issues and turnaround time.

The opportunity to have both tools available at the IRCCS San Raffaele Laboratory made it possible to implement the HTA with direct evidence; therefore, it was handy for the evaluation of accuracy and organizational aspects as it was possible to test the two analyzers in the context of the laboratory routine. This concept is also crucial in the case of already validated instruments, such as the Sebia Capillarys 2 Flex-piercing analyzer and Tosoh HLC-723G11 analyzer, which are included in the list of methods certified by the NGSP for HbA1c, and which are based on the two methods approved for analysis of the hemoglobin profile (13). In the experience reported, applying the HTA tool has made it possible to highlight how both analyzers respond equally to the clinical and organizational needs of the IRCCS San Raffaele Laboratory. However, the decision was made to adopt the Tosoh HLC-723G11 analyzer to replace the Sebia Capillarys 2 Flex piercing. The Tosoh HLC-723G11 showed adequate performance and rapid turnaround time in measuring HbA1c. This introduces a recently commercialized analyzer instead of an instrument that appears to be discontinued by the manufacturing company, whose management and maintenance risk is becoming complicated and expensive, with difficulties in supplying consumables.

### Generalizability of HTA findings

The HTA of the HLC-723G11 and Sebia Capillarys 2 Flex-piercing analyzers are initially contextualized within the specific operational and clinical settings of the IRCCS San Raffaele Hospital. This context includes a high throughput of laboratory tests, a diverse patient population with a prevalent incidence of diabetes and hemoglobinopathies, and an advanced technological infrastructure. Although HTA findings demonstrate clear benefits in terms of diagnostic accuracy and operational efficiency within the IRCCS San Raffaele Hospital, the question of their applicability to other laboratory medicine settings merits consideration. Laboratories with similar high-volume demands, patient demographics, and technological readiness are likely to experience comparable benefits from integrating the HLC-723G11 analyzer. However, caution should be exercised before generalizing these findings to laboratories with different organizational structures, such as smaller labs, those with lower test volumes, or those lacking the infrastructure to support advanced analyzers. In such settings, the specific advantages observed might not be as pronounced, and the cost–benefit ratio could differ significantly.

For laboratories considering the adoption of new analytical technologies, we recommend conducting a preliminary HTA that considers their specific organizational needs, resource availability, and patient profiles. Such an HTA should ideally be guided by principles similar to those used in our study, ensuring that decisions are grounded in robust evidence tailored to the local context.

## Conclusion

Implementing the culture and diffusion of HTA in Laboratory Medicine facilitates the reduction of the gap between technological innovation and stakeholders (policymakers, scientific-professional communities, health professionals, medical device industry, patient associations, and citizens): favoring the technologies that are more effective and with favorable clinical and organizational impact, without limiting oneself to the sole consideration of the relationship cost/benefit, means favoring the transition from the performance logic to the outcome logic (42).

A substantial advantage of the HTA is that it can and should be adapted to the specific context (43) to be able to support the choice of health technologies of decision makers at the micro (clinical managers), meso (managerial managers), and macro (policymakers) levels.

Laboratory specialists should adopt a critical attitude towards the method used to recognize its potential and limitations and evaluate, where appropriate and possible, a comparison with a different methodology. HTA is essential in Laboratory Medicine because the instruments are used for several years; therefore, the selection should not be based only on the analytical part and the costs, but a global approach is needed to allocate the resources efficiently. The costs of the laboratory (reagents and instrument) are, in general, limited compared to those of the entire diagnosis and treatment process, so it is crucial to consider the impact of the whole process on the organizational aspects and the patient journey.

Without the support of the assessment methods, the laboratory specialists do not adequately answer clinical questions, do not respect the required principles of appropriateness, and do not achieve the specific objectives of diagnosis, treatment, and prevention.

## Supporting information

Di Resta et al. supplementary material 1 Di Resta et al. supplementary material

Di Resta et al. supplementary material 2 Di Resta et al. supplementary material

## Abbreviations

HTA: Health Technology Assessment
DM: diabetes mellitus
CV: coefficient of variation
HPLC: High-performance liquid chromatography
CE: capillary electrophoresis
HbA1c: glycated hemoglobin

## Supplementary material

The supplementary material for this article can be found at http://doi.org/10.1017/S0266462324000497.
