# Supplementary material for: Links between accuracy and effectiveness of laboratory medicine equipment: use of the EUnetHTA core model to compare two analyzers by measuring HbA1c
Source: Int J Technol Assess Health Care. 2024 Dec 3;40(1):e67. doi: 10.1017/S0266462324000497 (PMC11703618; doi:10.1017/S0266462324000497)
Supplement: Di Resta et al. supplementary material 1 — Di Resta et al. supplementary material [file S0266462324000497sup001.pdf]

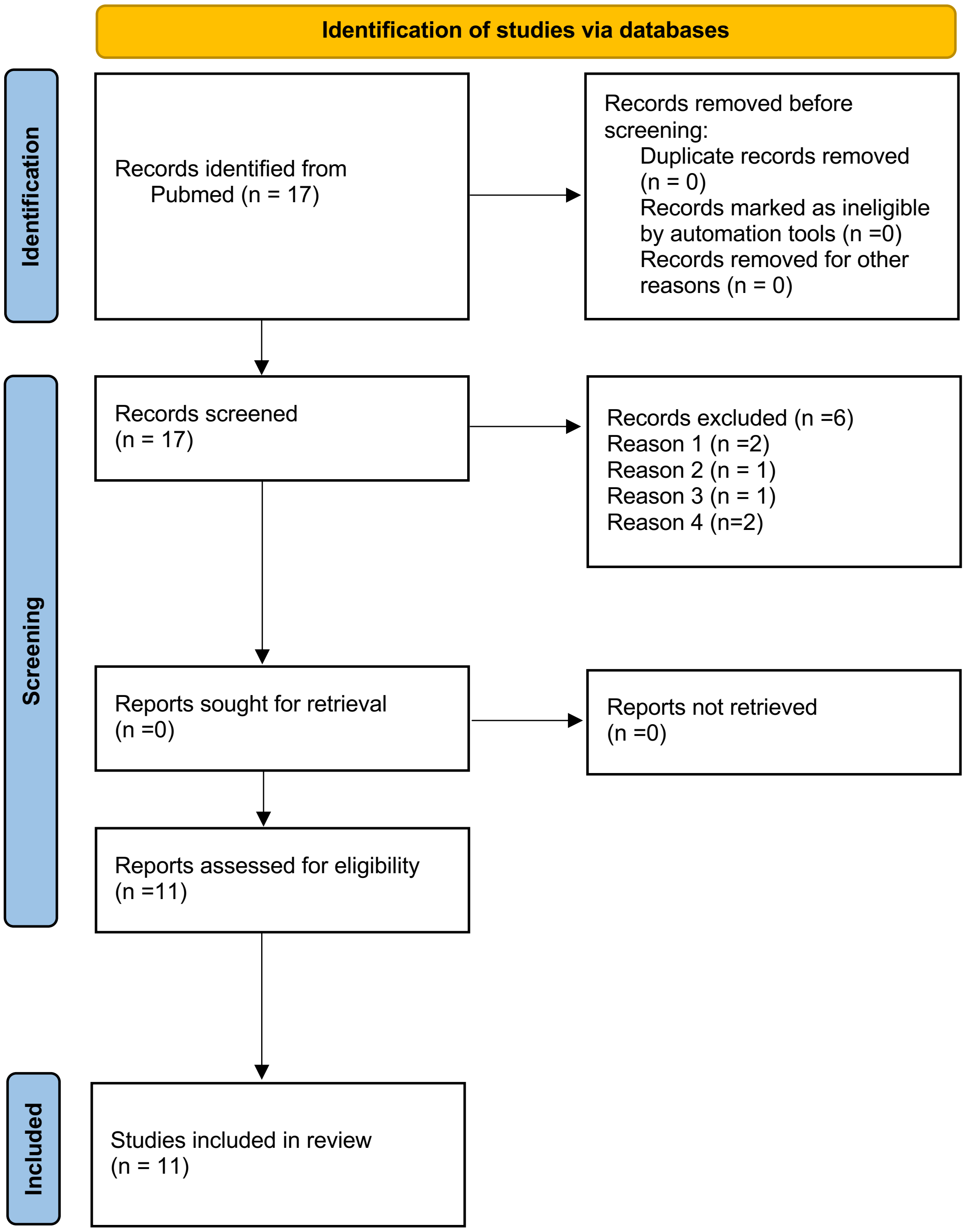

Reason 1: establishment of Reference Intervals
Reason 2: case report
Reason 3: analysis from in vitro erythroid cultures
Reason 4: Different diagnostic motivation
