# Supplementary material for: Links between accuracy and effectiveness of laboratory medicine equipment: use of the EUnetHTA core model to compare two analyzers by measuring HbA1c
Source: Int J Technol Assess Health Care. 2024 Dec 3;40(1):e67. doi: 10.1017/S0266462324000497 (PMC11703618; doi:10.1017/S0266462324000497)
Supplement: Di Resta et al. supplementary material 2 — Di Resta et al. supplementary material [file S0266462324000497sup002.pdf]

Health problem and current use of technology domain [12,18-22]

Target population	Target condition	Current managment of the condition
Affected by DM (5.9% of the Italian population) and at risk; individuals with suspected hemoglobinopathy (carried by 10% of the Italian population) and those undergoing neonatal screening	• DM and its complications: HbA1c is utilized for both diagnosis and therapy<br>• Hemoglobinopathies: Hemoglobin variants are employed in screening, diagnosis, and therapy	HbA1c is a useful measure of treatment efficacy, providing an integrated summary of circadian blood glucose over the preceding 6-8 weeks.

Technical characteristics domain [14,15,16,23]

	Sebia Capillarys 2 Flex-piercing	Tosoh HLC-723 G11
Type of Instrument	Automated analyzer based on capillary electrophoresis	HPLC with ion exchange
Quantitative Analysis	Hemoglobin fractions A, A2, and F; Detection of major variants S, C, E, and D	Separation of HbA1a, HbA1b, HbF, Labile HbA1c, Stable HbA1c, HbA0
Detection Method	Absorbance at 415 nm; Specific detection of hemoglobins	Absorbance at 415 nm/500 nm; High-resolution chromatograms
Results Expression	IFCC and NGSP units	Elution time differences for HbA1c values
Separation Considerations	Challenges of glycated HbA1c co-elution; Results expressed in both units	HbA1c eluted ahead of HbA, leading to potential overestimation/underestimation of HbA1c values

Safety domain [13,18,24]

Risk for patients	Hemoglobin Qualitative Abnormalities	Instrument Parameters	Safety of Healthcare Workers
The incorrect diagnosis and inaccurate quantification of HbA1c can adversely affect treatment decisions	The risk of unexpectedly identifying a hemoglobin variant and obtaining a false negative result may impact the assessment of hemoglobin structure	1. Certified by the NGSP<br>2. precision, linearity, and reliability parameters for HbA1c measurement and diabetes control	Disposal of biological materials and toxic waste.

Clinical effectiveness [23,25-35]

	Sebia Capillarys 2 Flex-piercing	Tosoh HLC-723 G11
Accuracy	CV < 2.5%, good linearity	CV < 2%, good linearity
	Both analyzers have been certified by the NGSP	
Correlation Between Analyzers	• Good correlation for HbA1c (R^2=0.972), decreases near diagnostic threshold (R^2=0.7032).<br>• Reasonable correlation for HbF (R^2=0.9274) and HbS (R^2=0.9667)<br>• Lower correlation for HbA2 (R^2=0.658)<br>• HPLC and CE identified as complementary methods for accurate hemoglobin variant quantification	

Organisational aspects domain [28,36]

	Sebia Capillarys 2 Flex-piercing.	Tosoh HLC-723 G11
Analyzers Characteristics	Perform analysis on whole or diluted blood (EDTA)<br>Record analytical sessions<br>Connect to the Laboratory Information System (LIS)<br>Usage by trained Laboratory Medicine personnel in a biomedical laboratory	
Turnaround Time	Provides 38 results per hour	HLC-723 G11 Variant Mode: 3’ for one sample, 12’ for 10 samples, 31’ for 30 samples.<br>HLC-723 G11 Standard Mode: 1’ for one sample, 7’ for 10 samples, 18’ for 30 samples
Analyzer Differences	at risk of obsolescence	a newly released instrument
	about a 70% difference in quotation between the two instruments	

Impact on the patient and society domain [31, 37-39]

Patients perception	Clinical Impact	Diabetes Management	Hemoglobinopathies Control
No direct perception of the laboratory test technology by the patients	1. Contribution to overall patient outcomes<br>2. comprehensive view of a patient's Hb profile for clinicians	It is crucial in public health management	critical in high-risk geographical areas

Ethical domain [39]

Ethical aspects	Benefit-harm balance
No differences	A detailed Hb profile assessment may uncover new variants, occasionally leading to critical hematological phenotypes when combined with beta and alpha chain defects.

Legal domain [40]

Essential levels of assistance (LEA)	Italian National Health Service (SSN)
Laboratory tests for DM and haemoglobinopathies are included in the LEA.	SSN is required to provide to all citizens ((DPCM, 2017)

**Color code:**	**No difference between analyzers**	**Difference between analyzers**
